# Method of determining loosely bound compounds of heavy metals in the soil

**DOI:** 10.1016/j.mex.2018.02.007

**Published:** 2018-03-01

**Authors:** Tatiana M. Minkina, Saglara S. Mandzhieva, Marina V. Burachevskaya, Tatiana V. Bauer, Svetlana N. Sushkova

**Affiliations:** Southern Federal University, 105, Bolshaya Sadovaya Street, Rostov-on-Don, 344006, Russia

**Keywords:** Method of determination of loosely bound metals compounds in the soil, soil samples, mobile forms of heavy metals, exchangeable form, complex form, specifically adsorbed form, parallel extraction, ammonium acetate buffer, ethylenediaminetetraacetic acid, hydrochloric acid

## Abstract

Method of determination of heavy metals loosely bound compounds in the soil was developed using three separate extractions. The group of loosely bound compounds of metals includes exchangeable, complexed, and specifically adsorbed forms. This method is available, rapid and not expensive. Extraction takes less than 24 h. Sample procedure preparation is simple, and the analysis consists of only three steps, which can be performed simultaneously. The parallel extraction gives reliable and reproducible results and provides a relatively complete idea of the metals mobility in the soil, their availability to plants, migratory capacity, and transformation.

• Method is suitable for a wide range of heavy metals and soil types. From the obtained data, the content of loosely bound compounds of heavy metals and the coefficients of metals mobility in the soil can be calculated.
• Method is suitable for estimation the microelement supply of uncontaminated soils. The content of elements in the 1 N CH_3_COONH_4_ extract characterizes the actual pool of elements, and their content in the 1 N HCl extract defines their potential pool in the soil.
• The coefficient of mobility (Km) is calculated to assess the contamination of soil with heavy metals. Estimation criteria of Km for Haplic Chernozem were developed.

## Graphical abstract

**Figure:**
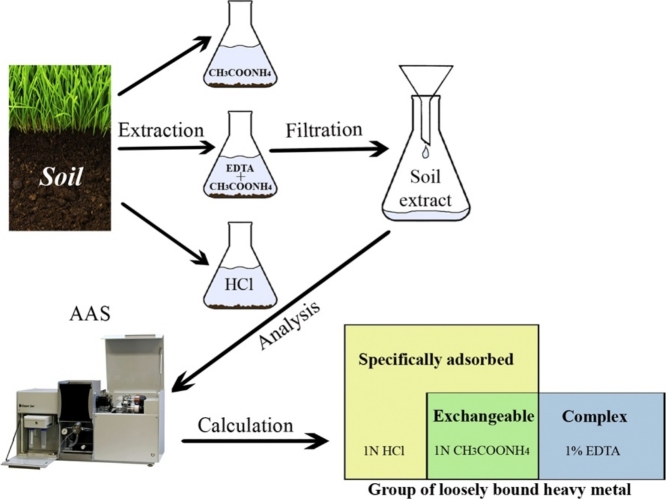

**Specifications Table**

**Table:** 

Subject area	*Environmental Science*
More specific subject area	*Soil science*
Method name	*Method of determination of loosely bound metals compounds in the soil*
Name and reference of original method	1. *Quantitative chemical analysis of soils. Procedure for measuring the mass fractions of mobile metals: copper, zinc, lead, cadmium, manganese, nickel, cobalt, and chromium in samples of soils, grounds, bottom sediments, and sewage sludge by flame atomic absorption spectrometry (PND F 16.1:2:2.2:2.3.78-2013, Federal Service for Environmental Control, Moscow, 2013, 21 p.).* 2. *Methodological recommendations for the determination of heavy metals in fodders and plants and their mobile compounds in soils. TsINAO, Moscow, 1993, 26 p.*
Resource availability	*Materials* • *Soil samples (analyzed soil portions) of 5* *g* • *White ribbon ashless filter papers* • *Glass funnels 60–80* *mm in diameter* • *Mortar, pestle, and cups in porcelain* • *Soil sieves with a 1-mm mesh* • *Conical glass flasks of 100* *mL* • *Glass jars of 100* *mL to store extracts* • *Measuring flask of 1* *L* • *98% CH_3_COOH* • *25% NH_4_OH* • *37% HCl* • *Ethylenediaminetetraacetic acid (EDTA)* • *Note: This list does not include any small generic laboratory equipment that are assumed to be available. Chemicals and other components can be used from any reliable company.* • *Distilled water* • *Analytical balance* • *Ionometer (pH-meter)* • *X-ray fluorescence spectroscopy* • *Atomic absorption spectrophotometer*

## Method details

For an objective assessment of the degree of environmental pollution, it is necessary to study not only the total content of pollutants in soil but also their loosely bound compounds. The diversity of the heavy metal compounds in soils makes it practically impossible and unfeasible to determine all the individual metal-bearing substances that are present in the particular soils. It is more efficient to determine the groups of heavy metal compounds differing in their mobility (migration capacity) in the given soils. The analysis of the metal group composition in the soil allows one to reveal the genetic specificity of the particular soils and to trace the effects of different factors on the soil properties. The main goal of the suggested method is the determination of the mobility of heavy metals in soils based on the data of their distribution between two major groups: firmly bound and loosely bound with the soil components. A group is a totality of metal compounds similar in binding strength to soil components and, hence, in migratory capacity and biological availability. The group of firmly bound compounds includes the metals strongly fixed in the structures of primary and secondary silicate and nonsilicate minerals, as well as the metals in difficultly soluble compounds and stable organic and organomineral compounds. The group of loosely bound compounds includes the metals associated with the soil particle surface with the organic and mineral soil components in the exchangeable and specifically sorbed states. Loosely bound compounds are the most important group of metals for the ecological point of view, as it enters plants and dispersed to environments. Hazardous ecological consequences of soil contamination with metals are related to these compounds [[1], [2], [3]]. The studies of the loosely bound compounds can assess not only the supply of nutrients to plants but also the degree of mobility and availability of toxic elements to living organisms, which is important to observe under anthropogenic pollution. The use of the proposed method allows to identify groups of elements with different mobility in specific soil conditions and to study in detail the soil-geochemical processes, which makes it possible to predict the behaviour of pollutants. The proposed method is fast, low cost and easily accessible to determine metals compounds available for plants in soil and representing the greatest environmental risks.

### Soil preparation

Soil samples were air dried in special room without chemical reagents at temperature (20–24 °С) and relative humidity 60–70%. The soil was dried to a moisture no more than 5–6% (the soil is dusty and dry to the touch). Soil samples were grinded up with a pestle and mortar after removing of large particles and plant residues. According Russian State standard, soil was sifted through a 1 mm sieve [4]. In Russia there are two standard methods of soil preparation: treatment with a 1 mm and 0.25 mm sieve [[5], [6], [7], [8]].

### Procedure

1. Preparation of solutions (extractants):
  a) To prepare 1 L of ammonium acetate buffer solution (1 N CH_3_COONH_4_), 108 mL of 98% CH_3_COOH and 75 mL of 25% NH_4_OH are added to 600–800 mL of distilled water. The solution can heat up; in the case of strong heating, the solution is left to cool before continuing. The pH of the prepared solution is measured with an ionometer; if the pH is higher or lower than 4.8, acetic acid or a 25% ammonium solution in water is added to adjust the pH value. Then, the solution is brought to 1 L with distilled water and mixed. The ammonium acetate buffer is accepted to extract elements available to the plants and serves to assess the availability of soils to these elements [6].
    The solution pH can vary depending on the object of study. A solution with pH 4.8 is used for neutral and weakly alkaline calcic soils, and a solution with pH 4.5 is used for acid and weakly acid soils in order to not shift the acid–base equilibrium in the soil [7]. Soil treatment with ammonium acetate buffer solution leads to partial desorption of metal ions from the weakest sorption centres and to the destruction of some metal complexes due to the complexing ability of the acetate ion. In the calcic soils, the use of the solution with pH 4.5 leads to a concomitant dissolution of metals bound to carbonates and some slightly soluble metal compounds and to the transfer it into the extract.
  b) To prepare a 1% solution of ethylenediaminetetraacetic acid (EDTA) in 1 N CH_3_COONH_4_, 10 g of EDTA is dissolved in 1 L of 1 N CH_3_COONH_4_ (preparation as at the preceding stage).
  c) To prepare 1 N HCl 82 mL of concentrate HCl is added to 500–600 mL of distilled water, and the solution is brought to 1 L with distilled water. The solution can heat up, in the case of strong heating, the solution is left to cool before continuing.
2. The following parallel extraction scheme was used to determine the contents of loosely bound compounds of heavy metals [[6], [9]]:
  a) A prepared soil sample (5.0 g) is placed in a conical flask. The minimum number of analytical replicates per sample is 3.
  b) The following solutions (50 mL each) are added to the prepared soil sample (5.0 g) with continuous agitation for 3 min.:
    i) an ammonium acetate buffer solution (1N CH_3_COONH_4_) with pH 4.8;
    ii) a 1% solution of EDTA in 1 N CH_3_COONH_4_;
    iii) 1 N HCl.
  c) The time of the extraction is 18 h. Extraction is performed at room temperature.
  d) The extracts are filtered with preliminary agitation for 3 min.
    All extracts need to keep in a closed glass jar at 4 °C until their analysis.
3. The heavy metals content in the extracts are measured with an atomic absorption spectrophotometer (KVANT 2-AT, Kortec Ltd, Russia). Calibration was carried out using standard solutions, and the instrument was adjusted to 283 nm, 325 nm, and 213.7 nm, for Pb, Cu, and Zn, respectively. For samples with very high levels of contamination, the digested soil solutions were diluted by a factor of 10 to bring them within the calibration range.
4. The total heavy metals content in the soil were determined by X-ray fluorescence (XRF) spectrometer («MAX-GV» spectroscan, SPECTRON, Russia). All samples were analyzed using the bulk mode for soil sample. Each sample was analyzed for 45 s per sample [10]. XRF is one of the simplest, most accurate and most economic analytical methods for the determination of the chemical composition of many types of materials. XRF analysis is more suitable than analysis with mixture of concentrated acids, aqua regia and so on. 26 elements including heavy metals can be determined simultaneously in a sample with XRF analysis. Relative errors between 1 and 10% are typical for trace element analysis. Results obtained with XRF are highly correlated with results from atomic absorption spectrometry but do not require labour and time-consuming sample digestion. XRF spectrometer could be employed to provide rapid in situ detection of the presence of toxic metals in soil samples.

The above procedure was also used to obtain a blank and control samples and all samples were blank-corrected. All laboratory tests were performed in triplicate.

### Calculation

**Fig. 1:**
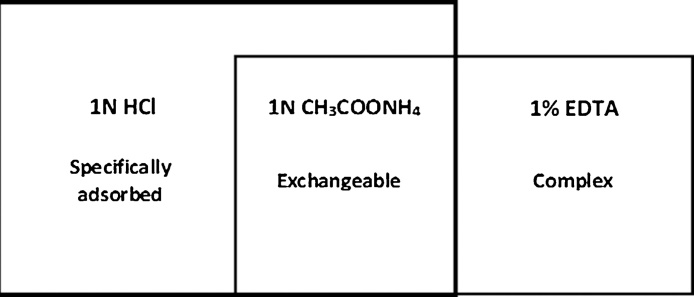
Group of loosely bound compounds of heavy metal in soils and their extractants.

The parallel extraction scheme of the loosely bound compounds of heavy metals and the corresponding extraction procedures are shown in Fig. 1. The metal compounds extracted with the 1 N CH_3_COONH_4_ are classified as exchangeable. The 1% EDTA in 1 N CH_3_COONH_4_ presumably extracts exchangeable heavy metals and those bound in the organometallic complexes; hence, the difference between the metal contents in the 1% EDTA in 1 N CH_3_COONH_4_ and 1 N CH_3_COONH_4_ extracts should characterize the content of metals in complexes with the soil organic matter.

The 1N HCl extracts specifically adsorbed metals together with exchangeable metals. A considerable part of the specifically adsorbed heavy metals is relatively loosely fixed by iron, aluminum, and manganese oxides and hydroxides and by carbonates. The contents of specifically adsorbed heavy metal compounds are calculated as the differences between their amounts extracted with 1N HCl and 1 N CH_3_COONH_4_. These compounds can be considered as a transitional group between loosely and firmly bound heavy metals.

The difference between the heavy metals content in 1 N CH_3_COONH_4_ and 1N HCl solutions is that the former displaces cations at the expense of participation of protons (pH 4.8) and ammonium ions; additionally, acetate ions bind the replaced cations in soluble complexes. As the concentration of the ammonium acetate buffer solution is high (1 mol L^−1^), the replacement function is largely due to ammonium ions. When 1N HCl is used as an extractant, part of the hydrogen ions is spent for the neutralization of carbonates and a part is spent for the replacement of exchangeable cations. Thus, the main effect of the 1 N CH_3_COONH_4_ solution is the replacement of exchangeable ions, whereas the main effect of 1N HCl is the acid hydrolysis of the soil sample. It can be supposed that the difference between the amounts of metals extracted by the 1N HCl and the 1 N CH_3_COONH_4_ characterizes the metal compounds bound with carbonates and oxides of iron and aluminum (specifically adsorbed metal compounds).

For the assessment of soil contamination with heavy metals, the coefficient of mobility (Km) was developed. Km is the ratio of the loosely bound (LB) to the firmly bound (FB) metal compounds in the soil:

Km = LB/FB

The contents of metals firmly bound (FB) with the soil organic and mineral components (immobile metals compounds) were calculated as the difference between the total contents of the metals and the group of loosely bound compounds. This coefficient of mobility (Km) can serve as a criterion of soil contamination and possible translocation of metals into plants. Estimation criteria of Km for Haplic Chernozem were developed: Km is 0–0.5 in no contaminated soil; 0.5–1.0 –contamination conditions.

## Method validation

Soil samples of Chernozem Haplic, Chernozem Calcic, Kastanozem Haplic [11] was taken on a monitoring sites from a 0 to 20 cm layer. The soil sample is made by mixing 5 initial samples collected at one monitoring site. The studied soils with different properties (Table 1) were contaminated under experimental conditions. Plastic pots with a closed drainage system were used. A 3 cm thick layer of washed glass beads was placed onto the bottom of 2 L plastic vessels for drainage. Then, 1 kg of soil sieved through a 2 mm sieve and mixed with dry copper (Cu), zinc (Zn) and lead (Pb) acetates were added to each vessel according to the experimental design. Soil incubation at room temperature and normal lighting continued for one year. Previous researches were shown [[12], [13]] this time is enough for transformation of added metals in the soil.

**Table 1:** Physical and chemical properties of studied soils, 0–20 cm layer.

Soil	Clay content (soil particle < 0.001)	C_org_, %	pH_H2O_	CaCO_3_, %	Exchangeable cations, cmol 100 g^−1^			Сation exchange capacity, cmol kg^−1^	Electrical conductivity, dS m^−1^	N-NO_3_, mg kg^−1^	P_2_O_5_, mg kg^−1^	K_2_O, mg kg^−1^
					Ca^2+^	Mg^2+^	Na^+^					
Chernozem Haplic	32.4 ± 1.5^a^	3.9 ± 0.2	7.5 ± 0.1	0.04	3.1 ± 0.1	0.6 ± 0.1	0.25 ± 0.01	39.5 ± 0.3	1.1 ± 0.5	0.25 ± 0.1	0.32 ± 0.1	2.28 ± 0.2
Chernozem Calcic	29.5 ± 1.8	3.6 ± 0.2	7.6 ± 0.1	0.2	2.9 ± 0.1	0.5 ± 0.1	0.06 ± 0.01	34.6 ± 0.2	1.0 ± 0.6	0.20 ± 0.1	0.16 ± 0.1	1.15 ± 0.2
Kastanozem Haplic	18.1 ± 1.4	2.6 ± 0.2	7.8 ± 0.1	0.6	2.0 ± 0.1	0.4 ± 0.1	0.01 ± 0.01	24.1 ± 0.1	0.9 ± 0.3	0.11 ± 0.1	0.12 ± 0.1	0.92 ± 0.1

a ±Standard deviation.

The experimental design included the control (original uncontaminated soil) and treatments with the addition of copper (Cu), zinc (Zn) and lead (Pb) at a rate of 55, 100, 300, 1000, 2000 mg kg^−1^. The experiment was established in triplicate (Table 2).

**Table 2:** Total content and loosely bound compounds of metals in the Chernozem Haplic.

Dose of metal, mg kg^−1^	Metal forms, mg kg^−1^			Total content, mg kg^−1^	Loosely bound compounds of heavy metals, % of the total content	Km
	exchangeable	complex forms	specifically adsorbed forms			
Cu						
Control (no added metal)	0.3 ± 0.01^a^	0.2 ± 0.01	2.0 ± 0.1	43.9 ± 1.5	6	0,1
55	3.3 ± 0.1	3.1 ± 0.2	19.1 ± 0.5	100.3 ± 2.0	25	0,3
100	13.1 ± 0.2	11.8 ± 0.9	26.1 ± 1.6	135.0 ± 2.2	38	0,6
300	26.3 ± 0.4	37.1 ± 1.1	52.4 ± 1.5	341.0 ± 3.5	34	0,5
1000	83.4 ± 1.0	173.0 ± 2.0	214.0 ± 3.0	1045.0 ± 10.0	45	0,8
2000	157.4 ± 1.5	337.0 ± 3.5	483.5 ± 4.7	2033.0 ± 20.0	48	0,9
LSD_0.05_	6.0	4.6	7.2	24.6		

Pb						
Control (no added metal)	0.6 ± 0.1	0.8 ± 0.2	2.2 ± 0.2	25.0 ± 1.5	14	0,2
55	2.2 ± 0.1	5.4 ± 0.5	5.3 ± 0.9	78.2 ± 1.8	16	0,2
100	7.5 ± 0.2	9.0 ± 0.8	20.1 ± 1.3	127.0 ± 2.5	29	0,4
300	19.0 ± 0.4	32.6 ± 2.9	70.9 ± 0.7	338.0 ± 3.5	36	0,6
1000	55.0 ± 1.0	157.0 ± 4.0	224.0 ± 3.0	1034.0 ± 11.0	42	0,7
2000	98.0 ± 1.2	579.3 ± 5.1	333.0 ± 3.5	2040.0 ± 19.0	50	1,0
LSD_0.05_	3.6	3.4	5.8	12.6		

Zn						
Control (no added metal)	0.4 ± 0.1	0.4 ± 0.01	5.6 ± 0.3	69.0 ± 1.7	9	0,1
55	1.5 ± 0.1	2.0 ± 0.2	49.2 ± 0.9	119.0 ± 2.5	44	0,8
100	7.0 ± 0.2	3.4 ± 0.6	65.0 ± 1.0	165.0 ± 2.0	46	0,8
300	51.0 ± 1.0	21.2 ± 1.2	99.1 ± 1.8	365.0 ± 3.0	47	0,9
1000	141.0 ± 1.6	137.0 ± 2.0	261.0 ± 4.0	1080.0 ± 9.0	50	1,0
2000	189.0 ± 1.8	305.0 ± 3.3	750.5 ± 7.3	2074.0 ± 21.0	60	1,5
LSD_0.05_	1.8	2.0	7.8	18.6		

a ±Standard deviation.

The salts of acetic acid were selected because of their good solubility and their capacity for quick and complete interaction with the soil mass. Contrary to the salts of mineral acids, acetates of heavy metals have some advantages: their hydrolysis is not accompanied by a sharp shift toward the strongly acid reaction, and acetate anions are the natural products of plant metabolism and cannot significantly change the nutrient regime of the soil.

The experimental data were statistically processed using EXEL2016. Experimental data are presented as means of three independent measurements (three replicate soil samples). Analysis of variance (Single factor ANOVA) was performed. Independent samples *t*-tests were used to compare any significant differences between the different extraction. Mean values were compared using the Least Significant Difference (LSD) test (P < 0.05), where the F-value was significant. The standard deviation (SD) are reported in Tables, where required.

The data obtained (Table 2, Table 3, Table 4) show that the lowest mobility in all investigated soils is typical for Cu (6–8% of the total content in unpolluted soil, and up to 54% in polluted soil). In polluted soil (1000 and 2000 mg kg^−1^), the group of loosely bound compounds increases up to 42–64% of the total content. Among all the studied soils of metals, there is a higher metal mobility in Chernozem Calcic and Kastanozem Haplic in comparison with Chernozem Haplic. Lower mobility in Chernozem Haplic depends on soil physical and chemical properties: texture, the content of organic matter and сation exchange capacity (Table 1). In uncontaminated soils, Km is 0.1–0.2 for all studied metals (Tables 2–4). Km depends on soil properties: the lowest Km is typical for Cu in Chernozem Haplic (from 0.1 to 0.9). Km in Kastanozem Haplic is higher than in others. This soil is least resistant to metal contamination. The highest Km among the studied metals is observed for Zn (up to 1.8), which indicates a strong pollution and a high ecological risk (Table 2, Table 3, Table 4).

**Table 3:** Total content and loosely bound compounds of metals in the Chernozem Calcic.

Dose of metal, mg kg^−1^	Metal forms, mg kg^−1^			Total content, mg kg^−1^	Loosely bound compounds of heavy metals, % of the total content	Km
	exchangeable	complex forms	specifically adsorbed forms			
Cu						
Control (no added metal)	0,3 ± 0.01^a^	0,3 ± 0.01	2,9 ± 0.1	45,0 ± 1.6	8	0,1
55	2,9 ± 0.1	2,6 ± 0.1	20,6 ± 0.7	93,4 ± 2.5	28	0,4
100	6,4 ± 0.4	8,7 ± 0.3	38,0 ± 1.2	138,7 ± 2.5	38	0,6
300	10,7 ± 0.5	46,2 ± 1.4	87,5 ± 1.6	354,0 ± 3.5	41	0,7
1000	81,0 ± 1.8	153,0 ± 1.9	254,0 ± 2.0	1043,0 ± 11.0	47	0,9
2000	134,0 ± 2.0	382,0 ± 4.0	549,0 ± 5.0	2048,0 ± 21.0	52	1,1
LSD_0.05_	5.1	3.5	5.4	21.3		

Pb						
Control (no added metal)	0,6 ± 0.1	0,3 ± 0.01	2,4 ± 0.1	23,5 ± 1.2	14	0,2
55	4,4 ± 0.4	3,1 ± 0.2	8,9 ± 0.3	76,0 ± 1.8	22	0,3
100	8,2 ± 0.6	6,8 ± 0.3	25,7 ± 1.0	125,0 ± 2.0	33	0,5
300	15,9 ± 1.0	36,7 ± 1.2	75,0 ± 1.5	335,0 ± 3.0	38	0,6
1000	62,0 ± 1.6	159,0 ± 2.0	258,0 ± 3.0	1029,0 ± 10.0	47	0,9
2000	112,0 ± 2.0	401,0 ± 4.0	561,0 ± 5.0	2037,0 ± 20.0	53	1,1
LSD_0.05_	4.4	5.3	3.9	11.2		

Zn						
Control (no added metal)	0,5 ± 0.1	0,3 ± 0.01	6,6 ± 0.3	65,0 ± 1.7	11	0,1
55	7,5 ± 0.3	5,9 ± 0.3	41,2 ± 1.1	121,0 ± 2.0	45	0,8
100	9,5 ± 0.4	6,8 ± 0.3	58,2 ± 1.3	159,0 ± 2.5	47	0,9
300	26,7 ± 0.9	32,4 ± 1.2	121,3 ± 2.0	360,0 ± 3.0	50	1,0
1000	79,0 ± 1.4	132,0 ± 2.0	334,0 ± 3.0	1075,0 ± 11.0	51	1,0
2000	226,0 ± 2.0	331,0 ± 3.0	698,0 ± 6.0	2068,0 ± 21.0	61	1,5
LSD_0.05_	2.2	4.9	9.6	12.8		

a ±Standard deviation.

**Table 4:** Total content and loosely bound compounds of metals in the Kastanozem Haplic.

Dose of metal, mg kg^−1^	Metal forms, mg kg^−1^			Total content, mg kg^−1^	Loosely bound compounds of heavy metals, % of the total content	Km
	exchangeable	complex forms	specifically adsorbed forms			
Cu						
Control (no added metal)	0,3 ± 0.01^a^	0,2 ± 0.01	2,4 ± 0.1	41,5 ± 1.5	7	0,1
55	3,1 ± 0.1	2,2 ± 0.1	21,4 ± 1.0	91,8 ± 1.9	29	0,4
100	8,9 ± 0.2	6,8 ± 0.1	31,7 ± 0.8	125,0 ± 2.0	38	0,6
300	22,0 ± 1.0	29,0 ± 1.2	92,0 ± 1.7	339,0 ± 3.0	42	0,7
1000	87,0 ± 1.7	108,0 ± 1.9	334,0 ± 3.0	1040,0 ± 10.0	51	1,0
2000	159,0 ± 2.0	257,0 ± 2.0	685,0 ± 7.0	2042,0 ± 19.0	54	1,2
LSD_0.05_	4.3	4.0	8.3	21.4		

Pb						
Control (no added metal)	0,5 ± 0.01	0,3 ± 0.01	1,9 ± 0.1	20,0 ± 1.0	14	0,2
55	4,6 ± 0.3	3,8 ± 0.1	12,5 ± 0.2	71,0 ± 1.6	29	0,4
100	12,0 ± 0.6	8,0 ± 0.5	25,0 ± 0.5	119,0 ± 1.9	38	0,6
300	31,0 ± 1.0	28,0 ± 1.0	71,0 ± 1.3	320,0 ± 3.0	41	0,7
1000	115,0 ± 1.5	128,0 ± 1.5	257,0 ± 2.5	1030,0 ± 9.0	49	0,9
2000	235,0 ± 2.5	324,0 ± 3.0	559,0 ± 5.0	2029,0 ± 20.0	55	1,2
LSD_0.05_	9.8	5.6	6.9	16.8		

Zn						
Control (no added metal)	0,4 ± 0.01	0,3 ± 0.01	7,7 ± 0.3	61,0 ± 1.5	14	0,2
55	3,9 ± 0.1	3,5 ± 0.1	43,1 ± 0.9	109,0 ± 1.9	46	0,9
100	11,8 ± 0.6	7,3 ± 0.1	58,1 ± 1.0	162,0 ± 2.0	48	0,9
300	41,0 ± 1.1	39,0 ± 1.0	107,0 ± 1.2	355,0 ± 3.5	53	1,1
1000	125,0 ± 1.5	141,0 ± 1.5	311,0 ± 3.0	1069,0 ± 11.0	54	1,2
2000	358,0 ± 3.0	367,0 ± 3.0	598,0 ± 6.0	2070,0 ± 19.0	64	1,8
LSD_0.05_	4.5	6.8	8.3	20.5		

## Additional information

The calculation of the complex-forming and specifically adsorbed metals compounds from the difference in the heavy metal contents in different extracts assume about their additivity. To verify this assumption, the procedure of sequential extraction of the metals with solutions of 1N CH_3_COONH_4_ and 1% EDTA and 1N CH_3_COONH_4_ and 1N HCl was used. Statistical analysis of the data showed that there were no significant differences between the results obtained by the method of sequential extraction and those calculated from the difference of the metal contents in parallel extracts (Table 5).

**Table 5:** Heavy metals determined with sequential extractions in Chernozem Haplic, mg kg^−1^ (*n* = 12).

Dose of metal, mg kg^−1^	Complex forms, determined with sequentially extraction (1% EDTA after 1 N CH_3_COONH_4_)	Specifically adsorbed forms determined with sequentially extraction (1 N HCl after 1 N CH_3_COONH_4_)
Cu		
Control (no added metal)	0.2 ± 0.01^a^	1.8 ± 0.1
55	3.3 ± 0.2	18.7 ± 0.3
100	9.9 ± 0.8	24.0 ± 1.4
300	36.0 ± 1.0	49.9 ± 1.2
1000	170.0 ± 3.0	216.0 ± 4.1
2000	332.3 ± 3.8	490.0 ± 5.0

Pb		
Control (no added metal)	0.6 ± 0.1	2.0 ± 0.1
55	4.8 ± 0.7	4.8 ± 1.0
100	10.3 ± 1.0	22.2 ± 1.5
300	36.0 ± 3.1	68.1 ± 1.0
1000	156.0 ± 3.2	225 ± 2.9
2000	571.6 ± 4.2	329.3 ± 3.3

Zn		
Control (no added metal)	0.5 ± 0.01	6.0 ± 0.5
55	2.4 ± 0.3	47.5 ± 0.7
100	4.3 ± 0.5	63.4 ± 1.1
300	19.1 ± 1.1	102.5 ± 1.9
1000	134.0 ± 2.3	266.0 ± 5.0
2000	309.9 ± 3.5	768.1 ± 8.7

The calculation method was also used by other authors to determine the content of firmly bound, difficultly available metal compounds in soil from the difference between the total metal content and its available (exchangeable and soluble) forms [[14], [15], [16]]. Other authors used the sequential treatment with a series of group extractants: these can be CH_3_COONH_4_ and 1 N HCl [[17], [18]]; EDTA and 1 N HCl [19]; Ca(NO_3_)_2_, CH_3_COONH_4_, and 1 N HCl [20]; and 1 M NH_4_Cl, CH_3_COONH_4_, and 0.1 M NaOH [21]. In the calculation method, available data on the metal compound sequential extraction in different soils can be used and expanded with the calculated parameters of metal contents in other (nonextracted) fractions.
